# The Quasi-Bound State as a Predictor of Relative Binding Free Energy

**DOI:** 10.1021/acs.jcim.5c00289

**Published:** 2025-05-20

**Authors:** Álvaro Serrano-Morrás, Yvonne Westermaier, Maciej Majewski, Xavier Barril

**Affiliations:** 1 Facultat de Farmàcia and Institut de Biomedicina, 16724 Universitat de Barcelona, Av. Joan XXIII, 27-31, Barcelona 08028, Spain; 2 Catalan Institution for Research and Advanced Studies (ICREA), Passeig Lluís Companys 23, Barcelona 08010, Spain

## Abstract

Relative binding free energy (ΔΔ*G*_bind_) predictions have become the main approach to evaluate the potency of a congeneric series of compounds. They are enabled by alchemical transformations coupled to free energy methods, tools that have become essential in drug design. Yet, they are computationally expensive and are limited to small compound sets and relatively simple transformations. The ever-increasing size of virtual screening databases demands faster methods to assess virtual hits. Here, we show that the structural robustness of protein–ligand complexes, measured as the free energy necessary to reach a quasi-bound state (Δ*G*_QB_) by Dynamic Undocking (DUck), is well suited to detect outliers in the structure–activity continuum (i.e., activity cliffs), which are particularly challenging for knowledge-based approaches. On different congeneric series of HSP90α, CDK2, and BACE1 inhibitors, we demonstrate that Δ*G*_QB_ can deliver excellent predictions. Despite the local nature of the measurement, these are in some cases comparable to the much more computationally demanding alchemical transformation methods. We find that for systems following a one-step dissociation model, Δ*G*_QB_ informs about the free energy of the transition state, allowing us to predict relative binding kinetics and, when the series present relatively constant on-rates, also ΔΔ*G*_bind_. This work has important implications for drug discovery, as it shows that within a well-defined applicability domain, high-throughput computational dissociation studies can deliver ΔΔ*G*_bind_ predictions that compare well with rigorous alchemical transformation methods at a fraction of the cost.

## Graphical abstract

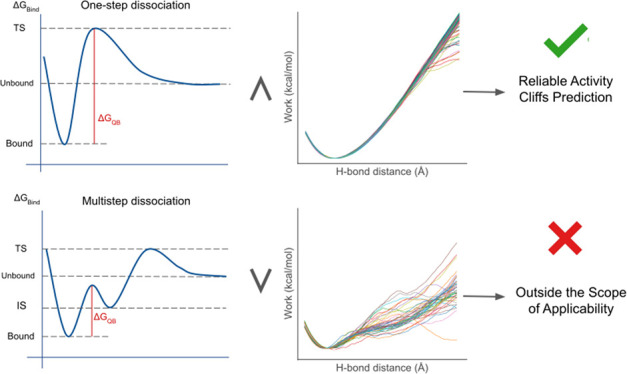

## Introduction

Predicting protein–ligand binding affinities is paramount for drug discovery but remains one of the major challenges of computational chemistry. The binding free energy (Δ*G*_bind_) of noncovalent complexes relates directly with the thermodynamic equilibrium constant (*K*_d_) and indirectly with the most common functional observables (*K*_i_, IC_50_, and EC_50_) that are often taken as a proxy of binding affinity. The importance of Δ*G*_bind_ has motivated the development of a wide spectrum of computational approaches to predict this property. Empirical (or trained) methods, also known as quantitative structure–activity relationship (QSAR) methods, rely on pre-existing data and have seen an important revival with the emergence of machine learning (ML) algorithms.^1^ However, they often assume a continuous relationship between binding affinity and chemical space. In this context, outliers that create *cliffs* in the structure–property landscape are particularly challenging.^2,3^ There are a plethora of structure-based methods to calculate Δ*G*_bind_, broadly classified as end-point (e.g., molecular mechanics with Poisson–Boltzmann and surface area solvation (MM/PBSA)), path-sampling (e.g., meta-dynamics or adaptive biasing force), or alchemical methods (e.g., thermodynamic integration (TI) or free energy perturbation (FEP)). These do not require pre-existing data, but end-point methods are rather inaccurate, while the rest are computationally expensive and offer variable performance.^4–6^

Relative binding free energy (ΔΔ*G*_bind_) calculations provide a useful shortcut when investigating a congeneric series of compounds that explore a similar configurational space.^7^

Hence, alchemical methods have become very popular for drug design.^9^ In the best case, they can be quite accurate (within 1 kcal mol^–1^ of the experimental value). However, accuracy decreases for challenging transformations (e.g., change in topology, net charge, multiple atoms, or protein conformation).^10^ Uncertainties can be assessed^11^ and reduced using multiple pairwise transformations per ligand.^12^ This increases the computational cost, which can be prohibitive for a large compound series. A rapid alternative to this method would be highly desirable, particularly in the hit-to-lead and early optimization stages of drug discovery, where thousands of analogs may need to be considered. Even if less accurate, it would be highly useful for drug design to predict activity cliffs (ACs; i.e., changes in *K*_d_ larger than 100-fold, corresponding to a ΔΔ*G*_bind_ > 2.73 kcal mol^–1^), thus helping to identify nonobvious leaps in potency.

Here, we investigate the potential of Dynamic Undocking (DUck) as a method to predict ΔΔ*G*_bind_. DUck was introduced as a postdocking filter in virtual screening campaigns, showing an 80 to 95% reduction of docking false positives.^13^ It is a particular implementation of steered molecular dynamics (SMD) used to efficiently evaluate the mechanical stability of protein–ligand hydrogen bonds. Leveraging the Jarzynski equation, the free energy of breaking native contacts is obtained from multiple short dissociation trajectories: The bound ligand is brought to a quasi-bound state, where an equilibrium between hydrogen bonds and water-mediated interactions takes place. The computed quasi-bound free energy (Δ*G*_QB_) provides information about the steepness of the ligands’ first dissociation barrier, which commonly acts as the main bottleneck during dissociation and defines the structural robustness of the protein–ligand complex.

The predictive power of DUck was unexpected because Δ*G*_QB_ is a local property with no correspondence to any macroscopic observable (Figure 1). Previous studies suggest that this characteristic reflects mostly on the dissociation kinetics, combining the effects of physical decoupling between H-bond partners during the rupture and resolvation of the ligand and binding cavity.^14^ Changes in the barrier steepness should not necessarily impact the thermodynamic equilibrium of a complex. As such, it cannot predict absolute binding free energies.^13^ However, previously, we have shown that (1) most protein–ligand complexes present high Δ*G*_QB_ values (i.e., they form mechanically robust hydrogen bonds),^14^ (2) the correct binding modes are generally also the ones with the highest Δ*G*_QB_ values,^15^ and, most surprisingly, (3) Δ*G*_QB_ quantitatively predicted ΔΔ*G*_bind_ of the ternary complex between Cereblon, Lenalidomide, and various mutant forms of Casein Kinase 1α.^16^ This prompted us to investigate whether Δ*G*_QB_ can rank-order congeneric series of protein–ligand complexes and, if so, why this magnitude would provide any insight into thermodynamic properties.

**Figure 1:**
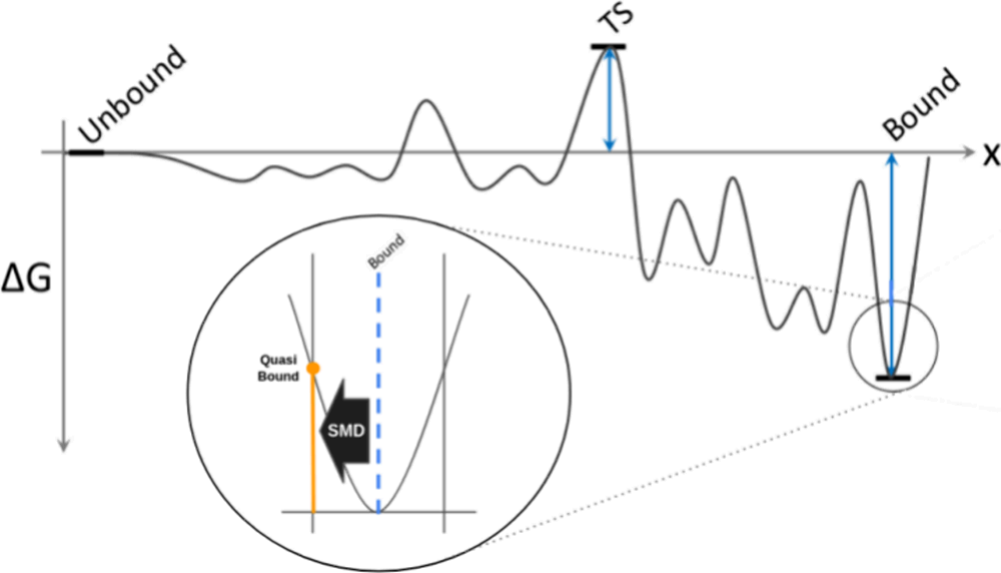
Schematic representation of the free energy landscape of a protein–ligand dissociation. Path-sampling methods aim at exploring this (presumably) complex landscape to predict the kinetic and thermodynamic observables. DUck introduces only a small perturbation to the bound state, yielding the free energy of the process. Contrary to expectation, this local property can, under certain conditions, inform about macroscopic observables.

## Results and Discussion

### Δ*G*_QB_ and Alchemical Transformations Are Comparable when Predicting Δ*G*_bind_ of Azole-Based HSP90 Inhibitors

We started by investigating the power of DUck in rank-ordering a set of isoxazole-, inverse isoxazole-, and pyrazole-based HSP90α inhibitors. HSP90α has several properties that make it ideal for our method. First, this HSP90α data set includes both thermodynamic and kinetic data (SPR and ITC data from Schmidtke et al.^8^ and personal communication by James Murray, Vernalis Ltd.). Second, the robustness of the essential interaction with D93 is an excellent predictor of binding and has been successfully used in DUck-based virtual screening.^12^ Finally, there is precedence with this system in using short SMDs for predicting and explaining the kinetic behavior of ligands.^17^

Figure 2 depicts the ligand core together with the different substitutions. Table 1 shows the substitution patterns for each of the nine ligands and the associated experimental data. In this core, 5-amides at R2 led to a more than 10-fold potency gain compared to the high-throughput screening hit, and switching then from pyrazoles to isoxazoles at X2 and adding a solubilizer group at R3 improved cellular potency by more than 3-fold.^18^

**Table 1:** Calculated ΔG_QB_ with Different Ligand Force Fields (PFROSST and GAFF) and Experimentally Measured *k*_on_ and *k*_off_ and Respective *K*_D_ Values for the HSP90α Vernalis Dataset^a^

ID	X1	X2	R1	R2	R3	*k*_on_ [M^–1^ s^–1^]	*k*_off_ [s^–1^]	*K*_D_ [nM]	Δ*G*_QB_ (PFROSST) [kcal/mol]	Δ*G*_QB_ (GAFF) [kcal/mol]
VER37655	N	NH	Br	Me	methoxy-phenyl	4.3 × 10^5^	2.2 × 10^–2^	51.2	10.9 ± 0.5	9.7 ± 0.1
VER 49181	N	NH	Br	CONHEt	methoxy-phenyl	4.6 × 10^6^	6.1 × 10^–3^	1.33	14.3 ± 1.3	13 ± 0.4
VER 49007	N	NH	Cl	CONH_2_	methoxy-phenyl	4.89 × 10^5^	2.3 × 10^–2^	47	14.3 ± 0.6	12.1 ± 0.9
VER 49008	N	NH	Cl	CONHMe	methoxy-phenyl	1.4 × 10^6^	1.1 × 10^–2^	7.86	14.8 ± 0.7	12.25 ± 0.8
VER 49009	N	NH	Cl	CONHEt	methoxy-phenyl	9.9 × 10^5^	6.9 × 10^–3^	6.97	15.05 ± 0.7	11.9 ± 1.0
VER 45862	N	O	H	Me	methoxy-phenyl	1.40 × 10^6^	1.49 × 10^–1^	(106)	16.1 ± 0.3	16.7 ± 0.4
VER 45861	N	O	Br	Me	methoxy-phenyl	1.28 × 10^5^	1.02 × 10^–2^	(79.7)	14.2 ± 0.6	14.4 ± 0.3
VER 50589	N	O	Cl	CONHEt	methoxy-phenyl	8.2 × 10^5^	4.05 × 10^–4^	0.494	15 ± 0.6	15.5 ± 0.6
VER 53003	O	N	Cl	CONHEt	methoxy-phenyl	1.16 × 10^6^	3.25 × 10^–4^	0.280	17.6 ± 0.6	16.2 ± 0.4

a Data were obtained from Schmidtke et al.,^8^ and via personal communication by James Murray, Vernalis Ltd.; values correspond to triplicates, except those shown in parentheses, which correspond to single measurements.

**Figure 2:**
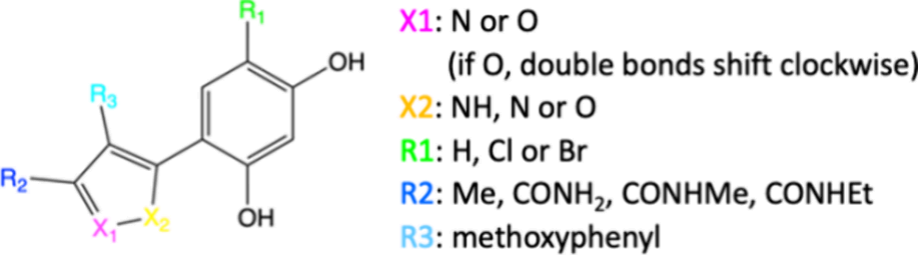
HSP90α inhibitors’ core and its different substitution points for the TI and DUck comparisons.

The activity changes of the isosteric replacements were correctly predicted using TI, with a correlation coefficient of 0.77 and a low mean absolute error, below 1 kcal/mol (Figure S1). Nonetheless, the changes by eliminating the ethylamide in R2 led to more pronounced errors. While the calculations of such challenging transformations could be mitigated by using more or longer windows, it would substantially increase the cost.

On the other hand, while DUck overestimated VER45862, which lacks a halogen at R1 necessary for hydrophobic complementarity, it provided excellent correlation for the remaining eight molecules, correctly predicting that amides at R2 and the isoxazole ring led to significant potency boosts. We evaluated the impact of the ligand force field during DUck calculations, comparing PFROSST and GAFF. While GAFF yielded overall lower Δ*G*_QB_ values, the ranking was maintained. Interestingly, the simulations with PFROSST showed better correlation with experimental *K*_D_ values (*r* = 0.69; ρ = 0.83), and GAFF with experimental *k*_off_ (*r* = 0.85; ρ = 0.76) (Figure S2). Despite the good correlations, the VER45862 outlier spotlights possible limitations of the method regarding ligands with higher conformational variability when bound and during dissociation.

Compared to alchemical transformations, DUck offers a major efficiency advantage, as each calculation is much faster and Δ*G*_QB_ is only calculated once per ligand (Table S1). It also predicts kinetic off rates better than equilibrium constants (Figure 3), regardless of the force field used. Yet, while alchemical transformations and other free energy methods have a sound theoretical foundation, this is not the case for DUck, which only samples configurations near the bound state and uses a small subset of the binding site (50 residues on average) to represent the receptor. Thus, it is particularly critical to determine its predictive power and applicability domain.

**Figure 3:**
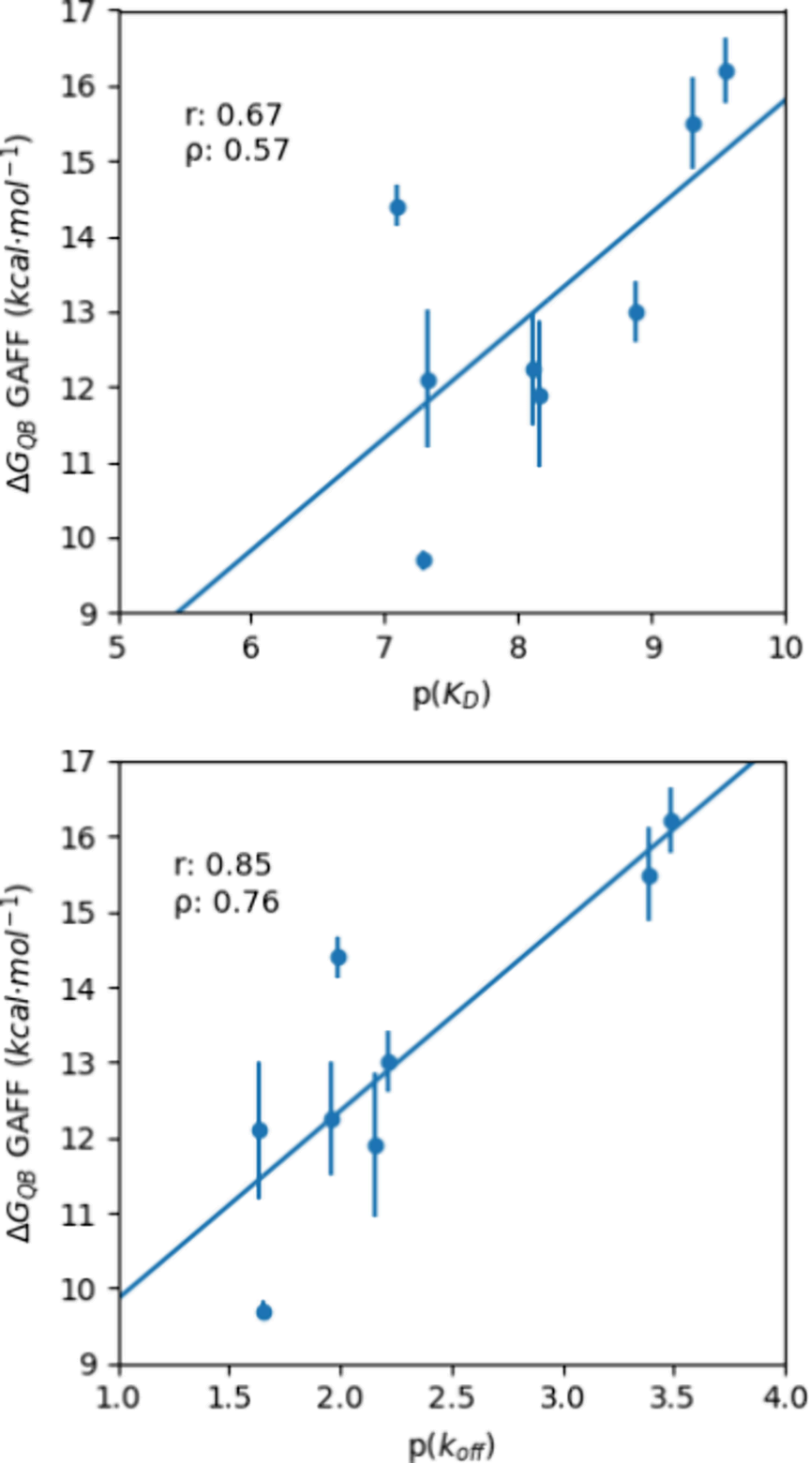
Correlations between the predicted Δ*G*_QB_ and the experimentally obtained p*k*_off_ and p*K*_d_, respectively. The shown dots correspond to the Vernalis ligands except VER45862, which was discarded.

### High-Throughput Δ*G*_QB_ Calculations Can Be Used to Accurately Predict the Formation of ACs

Taking advantage of DUck’s higher throughput, we investigated three systems for a total of 683 ligands. Estimating that quantitative ΔΔ*G*_bind_ predictions may fall outside the applicability scope for such a method. We thus ask whether Δ*G*_QB_ can predict ACs. ACs highlight structural motifs and groups of high relevance for a compound’s affinity, and their early detection is crucial in drug discovery. To corroborate the results obtained on Hsp90α, we first explore an extensive AC data set of the same system using data published by Kokh et al.^19,20^ The 93 inhibitors were paired by similarity and grouped by the receptor conformation they bind to: 13 to the loop conformation and 80 to the helical conformation, the latter leading to the opening of a cryptic site.^21^ The resulting 207 pairs showed a ratio of 1:10 of ACs, with only 18 pairs having affinity differences in the range of two to three orders of magnitude. Using DUck, we evaluated Δ*G*_QB_ for each HSP90α-inhibitor complex, finding that they range from 4 to 29 kcal mol^–1^. With a median Δ*G*_QB_ value of 21.9 kcal mol^–1^, they are skewed toward the higher value (Figure 4A) and match the top end of the distribution observed for a diverse set of protein–ligand complexes.^14^ As we plot the difference of Δ*G*_QB_ for each pair of compounds (ΔΔ*G*_QB_) vs the experimental relative binding free energies (ΔΔ*G*_bind_), we observe a trend where the largest changes in binding affinity also involve a large ΔΔ*G*_QB_ value (Figure 4B). While there is no straight correlation, the method is surprisingly good at detecting ACs. A scan of ΔΔ*G*_QB_ thresholds was performed via a receiver operator curve (Figure S9), selecting a ΔΔ*G*_QB_ of −5 kcal mol^–1^ as the best-performing threshold for such a task. Out of the 207 pairs, it identifies 29 possible ACs, 13 of which (45%) are correct. Conveniently, there are only a few false negatives (5; 2%). We obtain a Matthew’s correlation coefficient (MCC) of 0.52. For comparison, the well-known MM/GBSA scoring scheme has no predictive power (MCC = −0.13).

**Figure 4:**
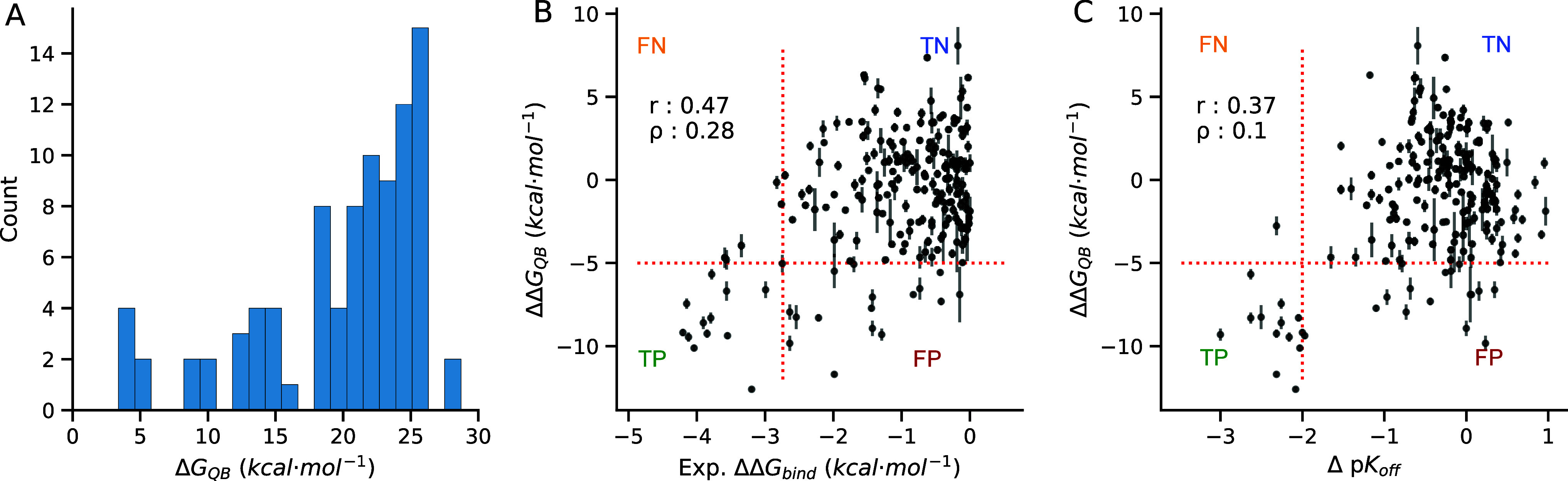
Prediction of activity cliffs from the HSP90α Kokh et al. data set. (A) Distribution of d*G*_QB_ values obtained from the Dynamic Undocking calculations in kcal/mol. Comparison between (B) ΔΔ*G*_QB_ vs ΔΔ*G*_bind_ and (C) ΔΔ*G*_QB_ vs Δp*K*_off_ for the 207 compound pairs, respectively. A ΔΔ*G*_QB_ threshold of 5 kcal mol^–1^ is set for predicting the activity cliffs with a ΔΔ*G*_bind_ cutoff of 2.73 kcal mol^–1^. The error bars correspond to the sum of standard deviations for each compared pair. Both thresholds are indicated as dotted lines. The prediction outcomes False Negative (FN), True Negative (TN), False Positive (FP), and True Positive (TP) are defined in the sections divided by those thresholds.

To better understand the method’s predictive power, we investigate its performance in predicting changes in kinetic off rates, which should be more directly related to the calculated magnitude. Indeed, cliff predictions based on *k*_off_ (Figure 4C) show a better correspondence than predictions based on *K*_d_ (MCC of 0.64 and 0.52, respectively). This indicates that DUck is well suited to guide the kinetic optimization of ligands, a task of considerable interest owing to the possible relationship of kinetic properties with *in vivo* efficacy.^22^

In practice, kinetic on-rates are often relatively constant within series,^23^ as exemplified in Table 1. This leads to an (imperfect) correlation between *k*_off_ and *K*_d_, which allows us to address the more general problem of predicting potency-based ACs.

Noticing that many false positive predictions involved specific ligands that are potent binders but present relatively low Δ*G*_QB_ values, we analyze their dissociation pathways, comparing them with the more standard case (high potency, high Δ*G*_QB_). We reproduced the random accelerated molecular dynamics (RAMD) simulations by Kokh et al.^19^ and monitored the intermolecular hydrogen bond used as a dissociation coordinate in DUck. The RAMD approach relies on applying a randomly oriented force to a ligand, which is updated given a displacement heuristic, forcing the ligand outside the binding pocket. On top of being a powerful tool to predict relative residence times (τ), it thoroughly explores the ligands’ dissociation pathways.^20^ We observed that, while most ligands follow a one-step dissociation (bound–unbound) with short, undetectable transition states (Figure 5A), some show a delay between rupture of the main H-bond and complete dissociation (Figure 5B). All ligands with a delayed dissociation had false AC predictions. Thus, we hypothesize that DUck can only deliver good predictions when ligands dissociate from the protein in one step. Under these circumstances, the quasi-bound state may be close to the real transition state (Figure 5C). Conversely, the appearance of intermediate (metastable) states makes the first barrier in the dissociation process irrelevant from a thermodynamic perspective (Figure 5D), rendering DUck ineffective. If confirmed, this would provide a rationale for the unexpected ability of DUck to predict ACs and, at the same time, it would allow us to define its applicability domain.

**Figure 5:**
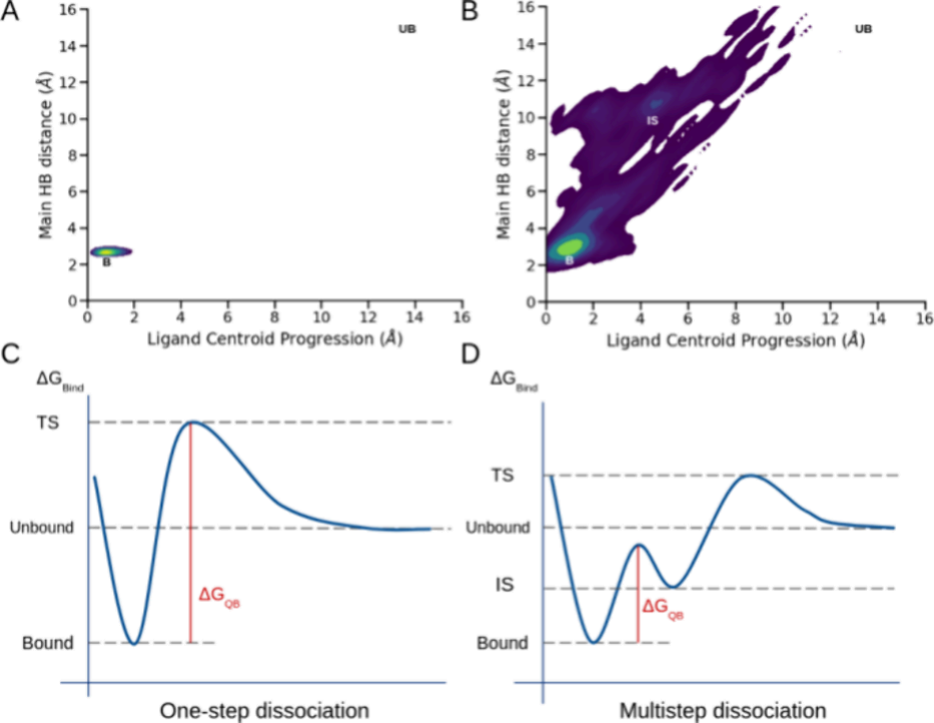
(A, B) Density representation of the ligand centroid movement (*x*) and the distance between the main interaction atoms (*y*) for the ligands 1g (A) and 2226756 (B) from the Kokh et al. data set. (C, D) Schematic representation of a one-step dissociation (C) and a multistep dissociation (D) free energy profile corresponding to what is seen in A and B, respectively.

### The Applicability of Δ*G*_QB_ to Predict ACs Is Defined by the Ligands’ Dissociation Mechanism

To confirm the correspondence between DUck’s efficacy and one-step dissociation, we analyzed a cyclin-dependent kinase 2 (CDK2) inhibitor data set using the same procedure. CDK2 is a well-studied kinase with more than 8000 activity entries in the ChEMBL database, of which 35% are IC_50_ values. We subdivided the CDK2 AC data set into four distinct congeneric series (CDK2-A, CDK2-B, CDK2-C, and CDK2-D), each using the scaffolds from the ligands in the PDB entries 2R3K, 1FVT, 2VTO, and 1JSV as a reference, respectively (Figure S4). This procedure minimized the structural differences between members of each set and identified the change responsible for each cliff. Combining the 497 CDK2 active ligands, we obtained 1164 pairs, 121 of which were ACs. The reference ligands’ binding mode was used as a template to generate binding poses for the analogs, assuming small substitutions will not induce a shift in the binding mode. Per series, a representative ligand pair was selected to explore the dissociation model *a priori* by τRAMD. The selection was guided by the structural similarity of the compounds with the rest of the series, prioritizing those with a resolved crystal structure. Inhibitors from series A and C showed metastable intermediate states, where the ligand no longer formed the H-bond with the hinge region but preserved the remaining interactions (Figure S4). By contrast, the representatives from series B and D featured a one-step dissociation, similar to Figure 5A, where rupture of the H-bonds resulted in immediate expulsion of the ligands from the binding site (Figure S5). Following the hypothesis generated for HSP90α, we predicted that DUck should have some predictive power for series B and D, but not for series A and C.

The complete set of ligands was then explored with DUck. Despite the high potency of the ligands in the data set, the distribution of Δ*G*_QB_ shows lower values for CDK2 (Figure S6) than for HSP90α (Figure 5A). Compounds from series A have predominantly labile interactions, followed by series D, while series B and C present more robust interactions (median Δ*G*_QB_ values of 6.6, 13.6, 13.4, and 9.7 kcal mol^–1^, respectively). The relatively high Δ*G*_QB_ values in series C contrast with the metastable states found during the τRAMD simulations. Then, we used the AC prediction protocol setup with the HSP90α data set, including the same predictive threshold and AC definition. With these parameters, the AC predictions of series B surpassed the HSP90α results, reaching an MCC of 0.6 and recovering 78% of the ACs. The predictive capabilities for the other series were worse: Series D has an MCC of 0.2, followed by series A and C, respectively, where no predictive capabilities were seen (i.e., MCC ≈ 0). The bad results from series C confirm that a steep quasi-bond barrier is an insufficient estimator of DUck’s predictive confidence. A one-step dissociation mechanism is also needed. On the opposite spectrum, series D shows that Δ*G*_QB_ can still have some predictive power (albeit mediocre) for series with low barriers, as long as there are no metastable states in the dissociation pathway.

To compare the performance of DUck to a standard rescoring method, we performed single-snapshot MM/GBSA calculations on every ligand, obtaining predicted Δ*G*_bind_. We then used the predicted ΔΔ*G*_bind_ values to classify the pairs, similarly to ΔΔ*G*_QB_. MM/GBSA is a well-established method for rescoring compounds from virtual screening campaigns,^24–26^ similarly to DUck. Despite being a faster method, MM/GBSA predictions show lower MCCs than for DUck, being close to 0 (random predictions) in most cases (Table S3). Nonetheless, the trend in predictive outcome depending on the scaffold series seems to be shared between both methods (Table S3). In contrast to DUck, MM/GBSA relies on end-point calculations, which should not be affected by the dissociation mechanism. This reveals underlying factors, intrinsic to each chemical series, which might be hindering the predictions.

### The Confidence in the ΔΔ*G*_QB_ Predictions Can Be Evaluated *A Priori* with a Simple Heuristic

Considering what has been observed in the other systems, we suggest the following two-step protocol to probe the applicability of Δ*G*_QB_ to predict ACs: First, systems with a robust main interaction (high Δ*G*_QB_ values) have shown better performance in the AC prediction (i.e., HSP90α and CDK2 series B) than labile ones. Thus, one should test representative compounds and proceed only if they reach high Δ*G*_QB_ values (e.g., >15 kcal mol^–1^). Second, a dissociation analysis must be performed for at least one or more representative compounds to check if dissociation happens in a single step. Testing a single representative assumes a common dissociation mechanism within a chemical series. Nonetheless, evaluating a subset of compounds might provide more confidence at a higher computational cost. We favor τRAMD, as it provides an enhanced sampling of the dissociation pathway without defining collective variables. However, alternative protocols could be equally valid. If those two conditions are met, we expect a high confidence in the AC predictions via their differences in structural stability.

As confirmation, we performed a prospective validation with the described steps. Due to recent insights into structural stability pointing to crystallized ligands having higher structural stability,^14^ we tested AC prediction on a new system, exclusively on ligands with a crystal structure. We used BACE1 as a test system, a pharmacologically relevant target with a less defined key interaction than the hinge interaction in kinases. From the set of 50 ligands from the PDBbind, forming 82 pairs, the proportion of cliffs to ligands was lower than in CDK2 due to the high scaffold diversity within the set. The location, number, and size of the changes have a strong impact (i.e., a series where all substitutions occur at the same substitution site will be able to form more cliffs than if they were dispersed, and the same is true for the size and number of substitutions). Despite the structural diversity, the main interacting groups were maintained across all ligands: an amide nitrogen interacts with the backbone oxygen of G291. Thus, we hypothesized that the dissociation mechanism is shared across the series, and the conclusions extracted with the reference compound should apply to the whole BACE1 data set.

Following the proposed protocol, we selected the ligand in the 2QZL PDB structure as a representative. From the dissociation trajectories obtained with τRAMD, we confirmed the one-step dissociation mechanism and a high residence time (Figure 6A). BACE1 has a loop closing the binding pocket, which opens during the unbinding event. This conformational change has slow kinetics but does not interfere with the ligand dissociation pathway, creating meaningful metastable states. When isolating the DUck chunk (i.e., reduced receptor representation), this loop is, however, removed. This is necessary due to the heavily restrained nature of the simulations. The Δ*G*_QB_ profile of the 2QZL ligand shows a very narrow distribution and a high free energy barrier of 18.4 kcal·mol^–1^ (Figure 6B). As the system met the two *a priori* conditions, we predicted ACs as expected, with high predictive power.

**Figure 6:**
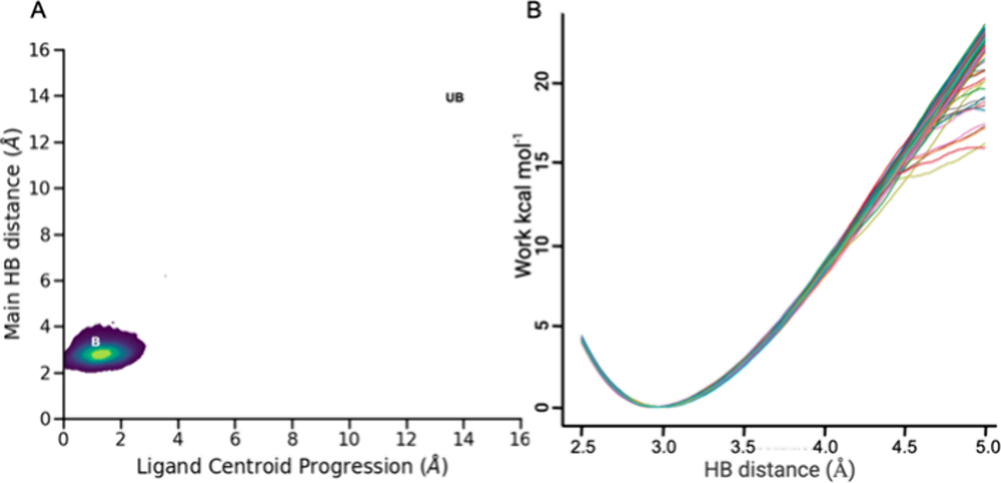
(A) Density of the ligand centroid movement (*x*) and the distance between the main interacting atoms (*y*) for the ligand of the PDB entry 2QZL during τRAMD simulations. (B) Work profile from the DUck simulations for the ligand of the PDB entry 2QZL.

BACE1 ligands presented a bimodal Δ*G*_QB_ distribution (Figure S7). Most compounds have a labile H-bond with values close to 4–5 kcal mol^–1^ and a small peak at very high quasi-bond free energies (15–20 kcal mol^–1^). Despite the lack of quantitative correlation between the experimental *K_i_* values and the Δ*G*_QB_ (Figure S7), the AC predictions yielded a high accuracy (MMC = 0.56), matching those on the HSP90α and CDK2 series B.

## Conclusions

Our results evidence that Δ*G*_QB_, despite being a local property of protein–ligand complexes, can be used to confidently predict ΔΔ*G*_bind_, a global property of the system. However, this is only true for ligands dissociating with (a) a steep barrier (early transition state) and (b) in a single step (no metastable states). Δ*G*_QB_ provides the barrier steepness, but the second condition is more difficult to predict. We suggest a simple protocol, using a reference ligand per chemical series. If the ligand’s Δ*G*_QB_ is high, we advise performing molecular dynamics studies to assess the dissociation mechanism. Here, τRAMD quickly showed a clear signal to distinguish between ligands following a one-step dissociation and those with more complex pathways; however, other meta-dynamics approaches can also be used.^27^ Using this approach, we have successfully predicted AC formation in three congeneric ligand data sets of different proteins. ACs remain a major challenge for virtual screening. On the one hand, while alchemical free energy calculating methods have shown promising results (see ref 7 and our TI results at the beginning of this study), they are too costly to be applied in a high-throughput fashion. On the other hand, data-driven approaches struggle due to the noncontinuous relationship between binding affinity and chemical space.^28^ In this scenario, DUck suggests a middle ground where reliable, albeit semiquantitative, predictions can be made in a high-throughput way, offering an ideal solution for systems within the scope of the method. It is noteworthy that in some cases, as we have shown for HSP90α, Δ*G*_QB_ can even be used for rank-ordering within congeneric series. Naturally, one can increase the quality of the prediction by using less drastic approximations (e.g., full system representation, slower dissociation, careful selection of the reaction coordinate). One important conclusion from this work is that theoretically rigorous path-sampling approaches have a great advantage when the system under investigation dissociates in a single step, with an early transition state. This appears to be a relatively frequent situation, and a situation that structure-based methods should take advantage of.

## Material and Methods

### Thermodynamic Integration on the HSP90 Vernalis Data Set

Single-transformation TIs were run with Amber16.^29^ “Single-transformation” means that the van der Waals and the charge transformations are carried out in one step using periodic boundary conditions. To avoid the boundary singularity effects close to the input and final structures, softcore versions of both the Lennard-Jones (LJ) and the Coulomb potentials were used. The softness of the LJ potential (scalpha) and the electrostatic potential (scbeta) were kept at their default values. Thus, the interactions among the “disappearing” atoms are unchanged, and any interaction involving at least one “(dis)­appearing” atom did not contribute to the average free energy change. Any shake constraint applying to bonds between common and unique atoms is being removed but kept for bonds between unique atoms.

The GAFF force field was used for all ligands, and the ff99SBildn force field for the protein. In each transformation, topology and coordinate files were generated for the initial and final conformations. Common atoms in both ligands were checked to have identical initial coordinates, and differing atoms were specified in the scmask. The crgmask contained atoms with the atomic partial charges set to zero. To switch on the free energy calculation, icfe and klambda were set to 1. clambda was set to five values between 0 and 1 (λ-values of 0.046, 0.230, 0.5, 0.769, or 0.953, respectively). ifsc was set to 1.

The relative binding free energy difference between ligand 1 (L1) and ligand 2 (L2) can be expressed as ΔΔ*G*_binding_ = Δ*G*_binding_ (L2) – Δ*G*_binding_ (L1) = Δ*G*_bound_ (L1 to L2) – Δ*G*_free_ (L1 to L2), where both the bound and the unbound, i.e., free ligand in solution, are considered in a thermodynamic cycle. The change in the area under the curve of d*V*/dλ is obtained via Gaussian quadrature. A 5-point Gaussian quadrature is calculated using the five λ-values. For each transformation, 10 ns were run at each λ-value for the complex and the free system. The cumulative average for each was then plotted against time to determine the convergence. The quality of the thermodynamic estimates and the correlation with experimental data improved when only considering the cumulative average of the last 4 ns (data not shown).

### System Preparation and Data Set Construction

For the rest of the study, three other data sets were prepared for ligands binding to HSP90α, CDK2, or BACE1 proteins, to detect ACs in those data sets. HSP90α ligands, their binding mode, and the receptor conformation they bind to were shared generously by Kokh.^19,20^ The data sets for CDK2 and BACE1 were compiled by extracting the ligands from crystal structures in the PDB.^30^ The CDK2 data set was expanded using compounds from ChEMBL with known activities.^31^

We generated the 3D structure of ChEMBL compounds and prepared them using LigPrep of the Schrödinger suite.^32^ Their binding poses were obtained with tethered docking calculations using rDock.^33^ We employed the maximum common substructure with the most similar compound from the PDB to select the tethered atoms. This was done assuming that small structural differences between the reference and docked compounds would not cause drastic shifts in binding mode. Nonetheless, those ligands that showed drastic changes in the binding mode during the MD sampling were removed from the series. The ligands with a disruption of the binding mode adopt a suboptimal position where the key hydrogen bond is very likely to dissociate. Therefore, the first indicator of a shift in the binding mode is an abnormally low work profile. Finally, the ligands were visually inspected to correct wrong protonation states and other artifacts from the preparation process.

Only ligands with reliable potency values were selected to retain high-quality reference experimental data. As such, we filtered out ligands without an exact potency value (e.g., having “<”, “>”, or “∼” symbols) in PDBbind,^34^ Binding DB,^35^ and the Binding MOAD.^36^ To broaden access to as many compounds as possible, the potency parameter selected was the IC_50_ instead of other values present in fewer compounds (i.e., *K*_d_ or *k*_i_). We are aware of the lack of consistency in the IC_50_ values between different assays and experimental conditions, and the effect it has on the validation of our predictions. The data sets were constructed with matched pairs with similar potency (noncliff) and high potency differences (cliffs). This was done to enable an analysis of the method’s performance in a realistic situation. ACs are defined by a high disparity in potency and a high structural similarity. Most authors agree on using a 100-fold difference (i.e., −2.73 kcal·mol^–1^ at 300 K) as a potency threshold to classify transformations as ACs. However, there is less consensus on the thresholds of structural similarity. Fingerprint-based numerical similarity allows the study of a wider variety of changes. Thus, we used MACCS key fingerprints coupled with the Tanimoto coefficient^37^ to maximize the available cliffs.

Overall, the HSP90α data set consisted of 93 HSP90α inhibitors (13 binding to the loop confirmation and 80 to the helical conformation), resulting in 207 pairs (18 activity cliffs and 89 noncliff pairs). The CDK2 series consisted of 497 active inhibitors, forming 1164 pairs (121 activity cliffs and 1043 noncliff pairs), with the series being very evenly populated. The 50 BACE1 inhibitors formed 82 pairs with only three activity cliffs.

### Dynamic Undocking

The DUck standard pipeline was used for each protein–ligand complex.^13^ Proteins were prepared using MOE-2016 ^38^ for correcting missing side chains, fixing protonation states, and adding terminal capping. Simulations were performed using a reduced receptor form (chunk). The chunk selection was done using the residues within 8 Å from the receptor’s atom involved in the hydrogen bond and then manually trimming out the external residues that do not interact with the binding pocket. Ligand parameters were obtained from the PFROSST force field,^1^ and the partial charges were calculated with AM1-BCC. To account for the plasticity of the HSP90α binding site, receptor coordinates were extracted from different PDB structures: 2VCI, 5J9X, 5OD7, and 5OCI for resorcinol loop binders, resorcinol helix binders, quinazolines, and imidazoles, respectively. In the case of CDK2, the PDB entry 1H1S was used to generate the protein conformation, and the H-bond to study was formed with the backbone of L83 in the hinge. The 1TQF PDB entry was used to model the BACE1 structure, where H-bonds with the backbone oxygen of G291 were studied. Each ligand was submitted to a DUck run with 120 cycles. Each iteration consists of a first cMD sampling step of 500 ps, and then an SMD stage for 500 ps.

Due to the disconnected nature of the chunk, protein heavy atoms were restrained with a harmonic force constant of 1 kcal mol^–1^ Å^–2^. The rupture of the key hydrogen bond during nonsteered simulations was restricted with a flat-bottom restraining potential for distances beyond 3 Å. The systems were minimized for 1000 steps, gradually heated from 100 to 300 K for 400 ps in four steps of 50 K each in the NVT ensemble, and then the pressure brought to 1 bar during a 1 ns simulation in the NPT ensemble. All equilibration and simulation steps were performed using a Langevin thermostat with a collision frequency of 4 ps^–1^. In each SMD, the H-bond partners were steered from 2.5 to 5 Å at a constant speed of 5 Å ns^–1^ for a total of 500 ps with a spring constant of 50 kcal mol^–1^ Å^–2^. The force needed to bias the system to maintain the constant speed was integrated into normalized work profiles (e.g., Figure 6B). As previously described,^13^ the selected work value (Wi) was the maximum along the work profile (quasi-bound state; i.e., the closest state where the investigated H-bond is broken), referenced to the minima that occurs around 3 Å (bound state, with an optimal H-bond). Quasi-bound free energies (Δ*G*_QB_) were calculated as the exponential average of the Wi, using the Jarzynski equality. Jarzynski’s equality (eq 1) relates the exponential average of work, obtained from simulating a nonequilibrium process repeatedly, directly with Δ*G*, where *k*_β_ corresponds to the Boltzmann constant, and *T* to the simulation temperature:

$$\Delta G_{\mathrm{QB}}=-k_{\beta }T\ln\left[\frac{1}{N}\sum_{i}^{N}e^{-W_{i}(k_{\beta }T{)}^{-1}}\right]$$ 1

The error estimation of Δ*G*_QB_ is calculated by resampling the 120 work trajectories in 100 random subsamples of 40 trajectories and extracting the standard deviation of the resulting resampled Δ*G*_QB_ populations. Based on the convergence of the Δ*G*_QB_ calculations shown in Figure S8, the 120 SMD cycles are enough to obtain converged free energy estimations. All simulations were performed with the AMBER16 CUDA version of PME.^39^

### τRandom Accelerated Molecular Dynamics

The protocol to perform the τRAMD simulations was reproduced from Kokh et al.^19,20^ All HSP90α ligands were tested, while only testing the ligand of the PDB entry 2QZL for BACE1 and 10 CDK2 inhibitors (CHEMBL409731, CHEMBL1234085, CHEMBL112136, CHEMBL333382, CHEMBL411426, CHEMBL271842, CHEMBL419534, and CHEMBL316809, as well as PDB entries 2R3K and 2C68). Using the whole (nonchunked) receptors, the systems were solvated in truncated octahedral TIP3P boxes with sodium chloride added to obtain charge neutrality. GAFF and ff14SB were used for the atom types and parameters for the ligands and proteins, respectively. The partial charges of the HSP90α ligands were provided by Kokh. They had been calculated via the RESP procedure from *ab initio* calculations at the Hartree–Fock level with the 6-31G*­(1d) basis set in GAMESS.^40^ The parameters for the HSP90α and BACE1 ligands were obtained from the DUck preparation (i.e., SMIRNOFF99 PFROSST force field and AM1-BCC-derived partial charges). The systems were minimized in AMBER16 with 500 steps of steepest descent and 1000 steps of conjugate gradient. They were then gradually heated to 300 K in 1 ns using harmonic restraints on the heavy atoms with a force constant of 50 kcal mol^–1^ Å^–2^. The restraints were decreased in five steps during equilibration (each of 1 ns with force constants of 50, 10, 5, 2, and 0.5 kcal mol^–1^Å^–2^). Then, a 10 ns sampling step was done without restraints. Equilibration and production were simulated at 300 K using a Langevin thermostat (with a collision frequency of 4 ps^–1^) and a Berendsen barostat at 1 bar, using 2 fs steps with hydrogen atoms constrained using the SHAKE algorithm.^41^ The equilibrated systems were transformed into NAMD format and reheated in steps of 10 K without restraints. A second equilibration was performed in NAMD^42^ in the NPT ensemble using a Langevin thermostat and Nosé–Hoover Langevin pressure control at 300 K and 1 bar, respectively. For each ligand, two snapshots of the equilibration were extracted as starting points for the τRAMD simulations.

We used the τRAMD tcl wrapper to perform random accelerated trajectories with a force of 16 kcal mol^–1^Å^–1^. The ligand center of mass (CoM) was checked every 100 fs. If the CoM of the ligand moved less than 0.025 Å, the direction of the force vector was updated. Simulations were stopped when the ligand’s center of mass moved further than 30 Å from its original position. A range of 15–50 dissociation trajectories was calculated depending on the τ convergence (given by the standard deviation bootstrapping). In addition to the ligand’s center of mass distance progression, the H-bond studied in DUck was also recorded.

### Evaluation of Activity Cliff Predictions

As a baseline comparison of DUck with other fast methods, we optimized rDock scores and performed single-point MM/GBSA calculations. For the rDock scores, the ligand coordinates (i.e., the crystal structures for HSP90α and BACE1 and the tethered docking poses for CDK2) were minimized with the minimize.prm rDock protocol. Then, SCORE.INTER was recorded as the docking descriptor. In parallel, the original poses were transformed into the Maestro format using the *structcat* command from the Schrodinger suite.^32^ The MM/GBSA calculations were performed using the Prime package in the Schrödinger Prime Suite, accounting for the ligand strain.^43^ The reported Δ*G*_bind_ was recorded as the MM/GBSA descriptor. Differences in SCORE.INTER and Δ*G*_bind_ were used to predict ACs, in the same way as Δ*G*_QB_ in DUck.

Areas under the ROC curve were calculated from the thresholds following the rank order of each scoring method. We selected −5 kcal mol^–1^ as the threshold (Figure S9). Due to the imbalanced nature of the data set (15% cliffs vs 85% noncliff pairs), performance was evaluated using the Matthews correlation coefficient (MCC), calculated as shown in eq 2.^44^

$$\mathrm{MCC}=\frac{\left(\mathrm{TP}\times \mathrm{TN})-(\mathrm{FP}\times \mathrm{FN}\right)}{\sqrt{\left(\mathrm{TP}+\mathrm{FP})(\mathrm{TP}+\mathrm{FN})(\mathrm{TN}+\mathrm{FP})(\mathrm{TN}+\mathrm{FN}\right)}}$$ 2

## Supplementary Material





## Data Availability

The data underlying this study are available in the published article and its Supporting Information. Input coordinates for ligand and receptors, together with DUck and τRAMD execution scripts, are available at https://zenodo.org/records/14888646.

## Notes

Collection of Δ*G*_bind_, Δ*G*_QB_, and AC predictions (XLSX)
TI and Δ*G*_QB_ values, initial structures, and AC prediction success rates for HSP90, CDK2, and BACE1 ligands with a detailed description of the methodology used (PDF)
