# Supplementary material for: The Quasi-Bound State as a Predictor of Relative Binding Free Energy
Source: J Chem Inf Model. 2025 May 20;65(11):5544–52. doi: 10.1021/acs.jcim.5c00289 (PMC12164264; doi:10.1021/acs.jcim.5c00289)
Supplement: Supplementary file 2 [file ci5c00289_si_002.pdf]

Supporting Information

The Quasi-bound State as a Predictor of Relative Binding Free Energy

Álvaro Serrano-Morrás[1], Yvonne Westermaier[1], Maciej Majewski[1], Xavier Barril[1,2]*

[1] Facultat de Farmàcia and Institut de Biomedicina, Universitat de Barcelona, Av. Joan XXIII, 27-31, 08028 Barcelona, Spain.
[2] Catalan Institution for Research and Advanced Studies (ICREA), Passeig Lluís Companys 23, 08010 Barcelona, Spain.
* barrilslab@gmail.com

Supplemental Tables and Figures

Table S1. Comparison of computational details and resources per method.

Table S2. Comparison of experimental $\Delta\Delta G_{bind}$ rank order with rank orders of $\Delta\Delta G_{bind}$ by thermodynamic integration and the total work needed to reach the quasi-bound state by Dynamic Undocking.

Table S3. Matthew Correlation Coefficients of the activity cliff predictions using different descriptors.

Figure S1. $\Delta\Delta G_{bind}$ predictions by Thermodynamic Integration.

Figure S2. Correlation between the predicted ΔG_{QB} and the experimentally obtained pk_{-1} and pK_d.

Figure S3. Representation of the interaction between the VER49009 inhibitor and the ASP93 of HSP90α.

Figure S4. Structural representatives of the CDK2 activity cliff chemical series.

Figure S5. Density profiles of the dissociation simulations for the structural representatives of the CDK2 activity cliff chemical series.

Figure S6. Distribution of ΔG_{QB} values for the four CDK2 congeneric series.

Figure S7. Relationship between K_i and predicted ΔG_{QB} of BACE1 inhibitors.

Figure S8: Convergence of the ΔG_{QB} calculations based on the number of SMD replicas.

Figure S9. Receiving operating curve of the activity cliff predictions using the HSP90α dataset.

Figure S10: Correlation between $\Delta\Delta G_{QB}$ and $\Delta\Delta G_{Bind}$ predicted by TI for the Vernalis HSP90α dataset.

Figure S11. Prediction of MMP activity cliffs from the HSP90α Kokh et al. dataset.

Supplemental Tables and Figures

Table S1: Comparison of computational details and resources per method. The values shown correspond to the computational details used in this manuscript, accounting for 60 steered molecular dynamics cycles per ligand for the dynamic undocking simulations and the 10 ns per window for the thermodynamic integration, with 7 windows per transformation. Compute times were obtained from K80 NVIDIA GPU cards.

Method	Dynamic Undocking	Thermodynamic Integration	MM/GBSA	τ Random Accelerated Molecular Dynamics
Average accumulated simulation time to full convergence	75 ns per ligand	280 ns per transformation	Single snapshot	20-180 ns per ligand (average of 160 ns)
Average size of the receptor	50 residues	300 residues	300 residues	300 residues
Average transformations per ligand comparison	-	2.2	-	-
Average wall clock time per ligand	4 h	215 h	0,027 h (1,6 min)	520 h
Ligands/Transfor-mations evaluated	683 ligands	11 transformations (5 ligands)	683 ligands	101 ligands
Total wall clock time	2800 h	1100 h	18,4 h	52500 h
Resources	1 GPU:CPU	1 GPU:CPU	1 CPU	1 CPU

Table S2: Comparison of experimental $\Delta\Delta G_{bind}$ rank order with rank orders of $\Delta\Delta G_{bind}$ by thermodynamic integration (TI) and the free energy needed to reach the quasi-bound state (total ΔG_{QB}) by Dynamic Undocking (DUck). The rank order of measured and predicted $\Delta\Delta G_{bind}$ was extracted from Figure 3. The total ΔG_{QB} for each transformation path is the sum of the respective approximate ΔG_{QB} values from Table SI.1.

Transformation path	Rank order of measured $\Delta\Delta G_{bind}$	Rank order of predicted $\Delta\Delta G_{bind}$ (TI)	Rank order of total ΔG_{QB} (DUck)
VER49181 – VER49009	3	3	5
VER49181 – VER49009 – VER49008	4	5	4
VER49181 – VER49009 – VER49008 – VER49007	5	4	2
VER49181 – VER49009 – VER50589	2	1	3
VER49181 – VER49009 – VER50589 – VER53003	1	2	1
How well do the predictions match the ranking of measured $\Delta\Delta G_{bind}$?		Good match: Transformations to isoxazole and inverse isoxazole were always better predicted than if the pyrazole was kept.	Rather good match: Transformations to inverse isoxazole were predicted better than to isoxazole, but not always predicted better than if the pyrazole was kept.

Table S3: Matthew Correlation Coefficients of the activity cliff predictions using different descriptors. In the Dynamic Undocking (DUck) column, the $\Delta\Delta G_{QB}$ was employed. For MM/GBSA and rDock, the respective $\Delta\Delta G_{bind}$ and SCORE.INTER was used. The differences in molecular weight were compared as a null hypothesis descriptor.

**Systems**	**DUck**	**MM/GBSA**	**rDock**	**Molecular Weight**
HSP90 (Kokh dataset)	0.527	-0.121	-0.133	-0.006
CDK2-Series A	0.075	-0.287	-0.226	0.090
CDK2-Series B	0.616	0.163	0.136	0.077
CDK2-Series C	0.065	-0.040	-0.187	0.021
CDK2-Series D	0.281	-0.080	0.092	0.078
BACE1	0.556	0.250	-0.160	-0.036

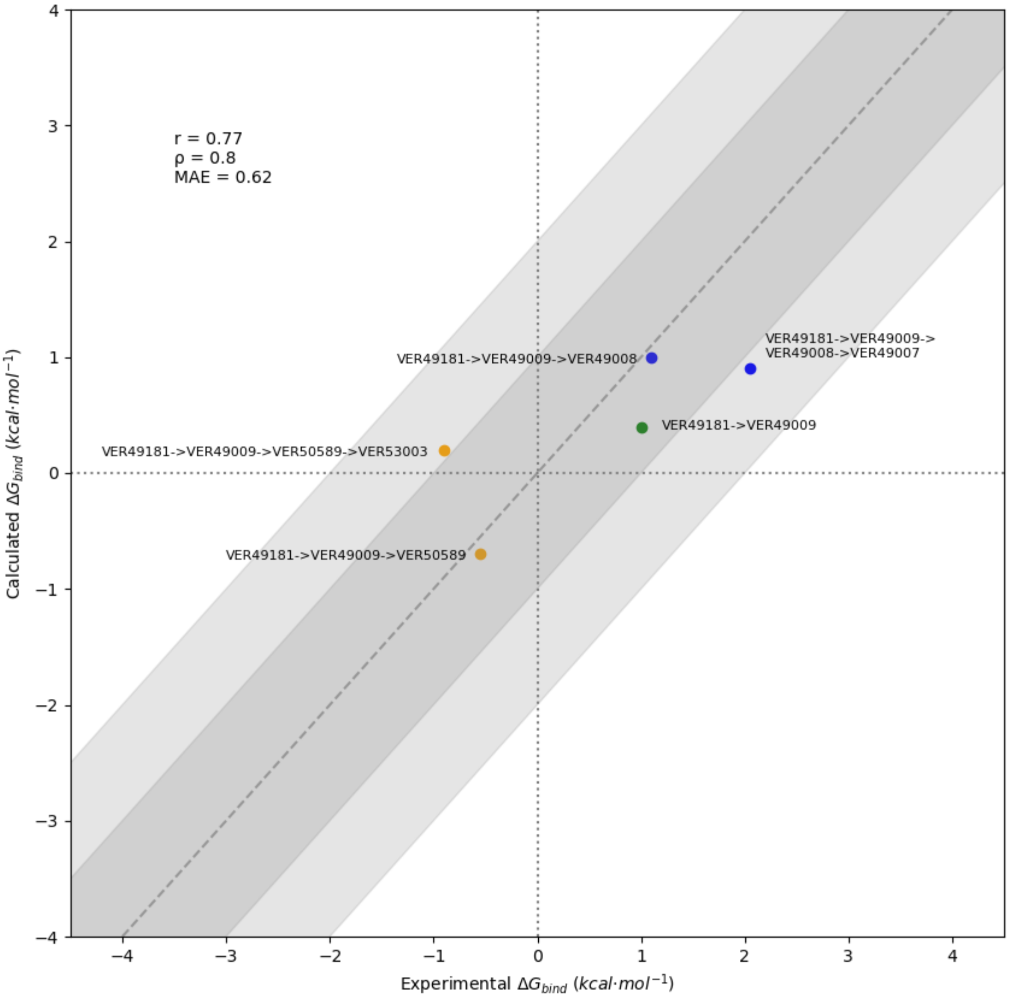

Figure S1: $\Delta\Delta G_{bind}$ predictions by Thermodynamic Integration. The predictions are within 2 kcal mol^{-1} of the theoretical perfect correlation, except for transformations changing the size of the R2 substituent drastically (VER49181 vs. VER37655, VER45861, and VER45862; not shown). Transformations to isoxazole, inverse isoxazole (orange circles), or pyrazoles (other colors) are in distinct areas of the Figure. One can distinguish between transformations of the core and the R-groups. Values on the x-axis are relative measured $\Delta\Delta G_{bind}$ values in kcal mol^{-1}, and values on the y-axis are relative calculated $\Delta\Delta G_{bind}$ values in kcal mol^{-1}. For both, VER49181 served as the reference.

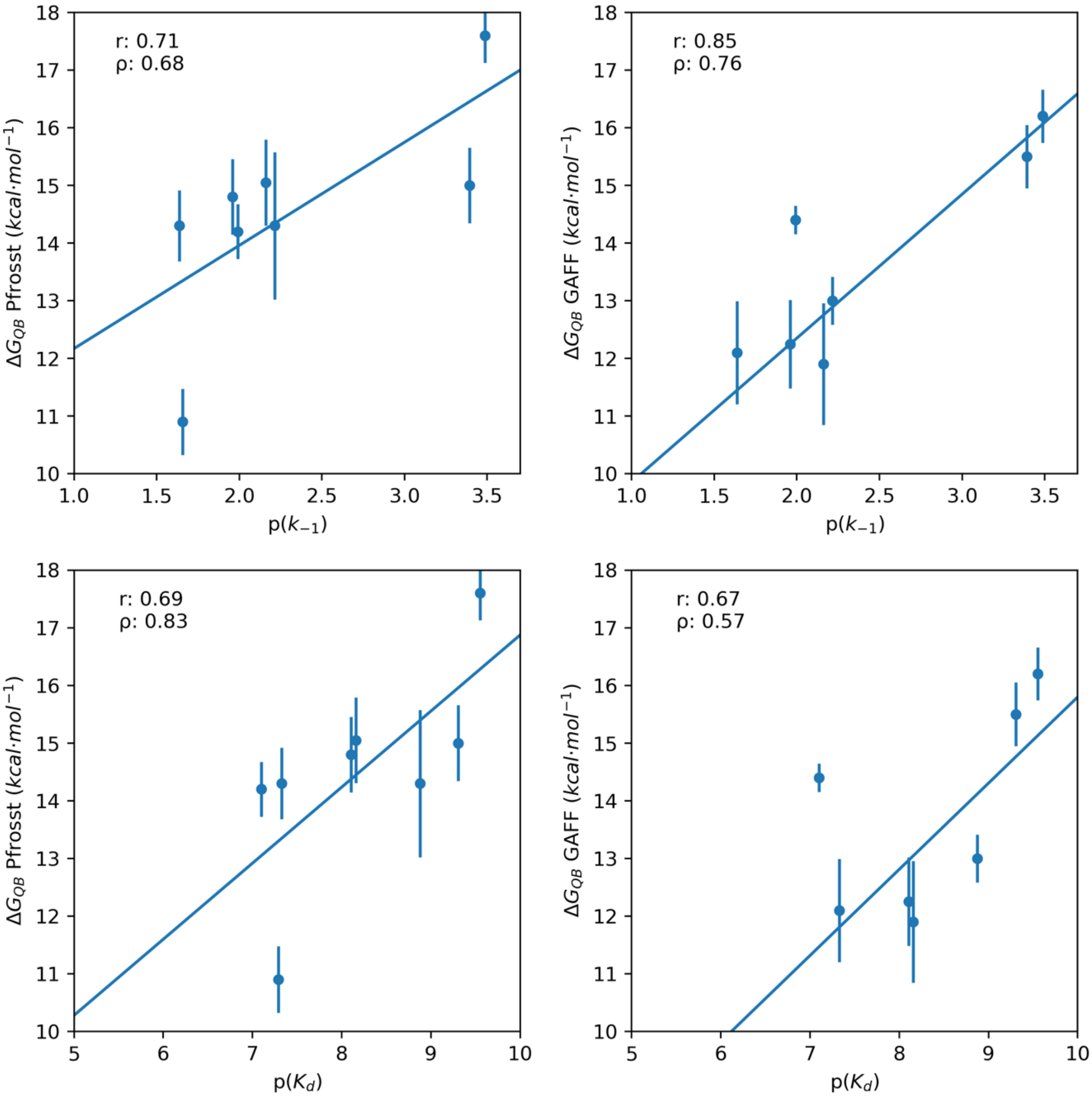

Figure S2: Correlation between the predicted ΔG_{QB} and the experimentally obtained pk_{-1} and pK_d. The shown dots correspond to the VERNALIS ligands except VER45862, which was discarded. The blue line corresponds to the linear model of the comparisons, the r to the Pearson correlation coefficient, and the ρ to the Spearman rank coefficient.

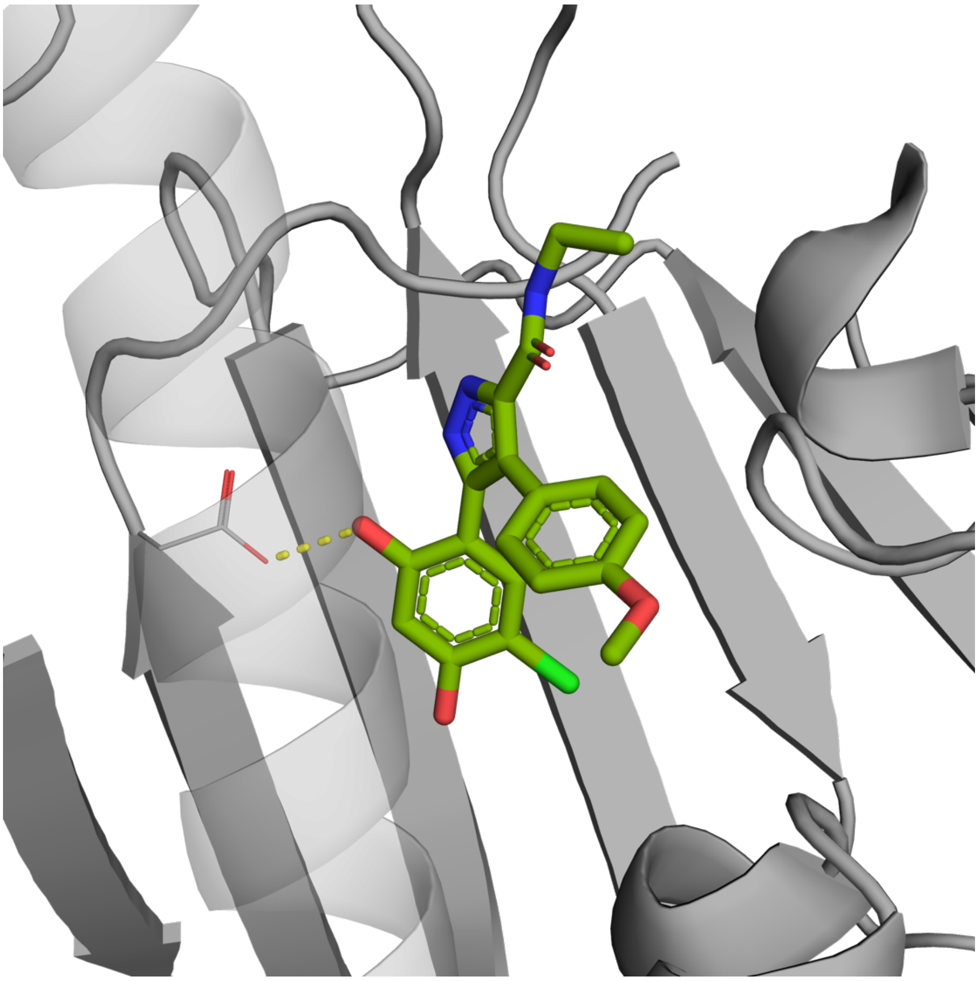

Figure S3: Representation of the interaction between the VER49009 inhibitor and the ASP93 from HSP90α.

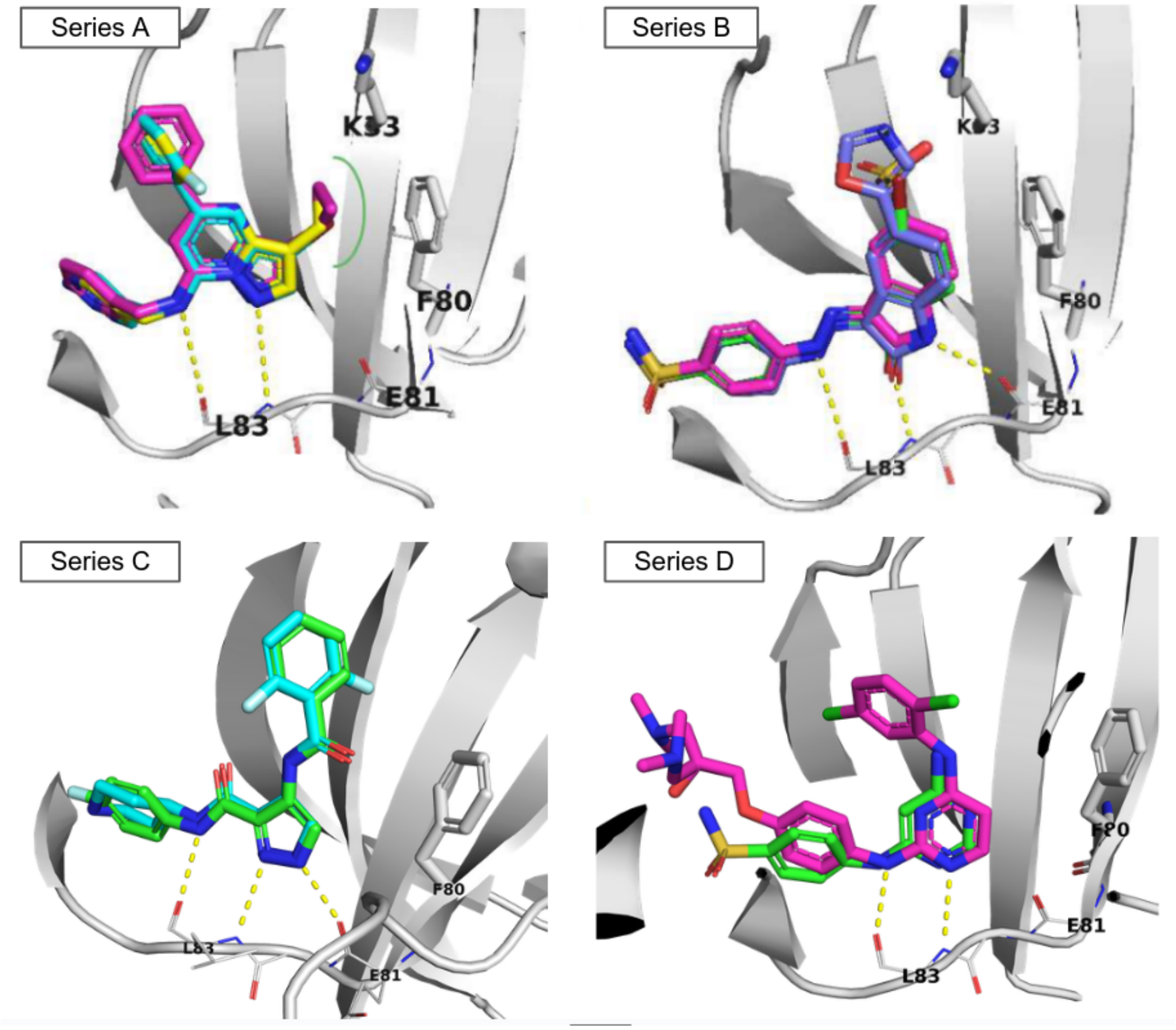

Figure S4: Structural representatives of the CDK2 activity cliff chemical series. For series A, the ligands of the 2R3K, 2R3M, and 2R3N PDB entries are shown with yellow, magenta, and cyan carbons, respectively. For series B, the activity cliffs of ligands CHEMBL269827 (purple) and ChEMBL412091 (magenta) are shown. Ligands of the 2VTO (green carbons) and 2VTQ (cyan carbons) PDB entries show the series C scaffold. For the CDK2 series D, the PDB entries 1H01 (light blue carbons) and 1OIR (yellow carbons) are represented.

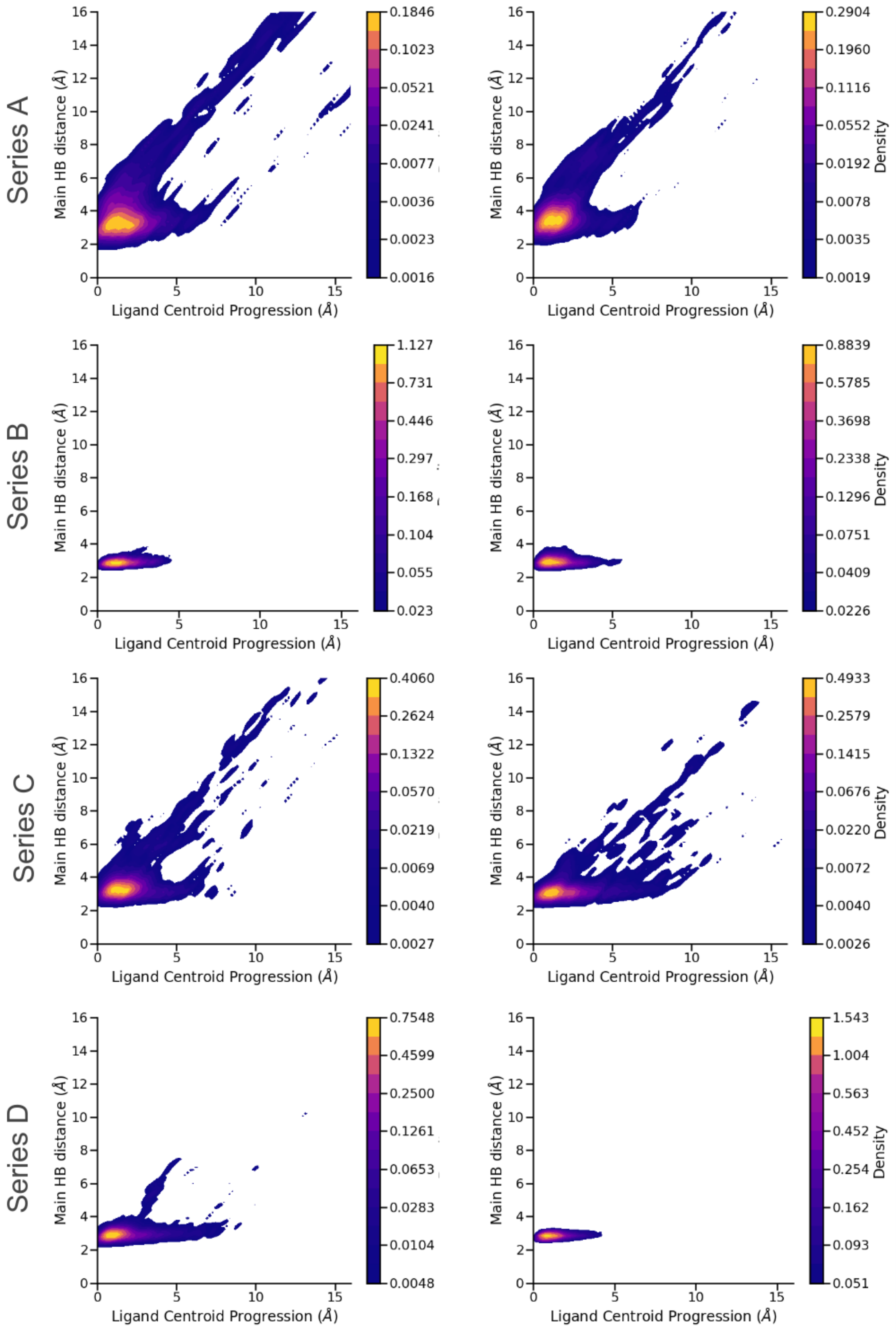

Figure S5: Density profiles of the dissociation simulations for the structural representatives of the CDK2 activity cliff chemical series. From left-to-right and top-to-bottom, the ligands correspond to the series A ligands of the 2R3K and 2R3N PDB entries; for series B, to CHEMBL269827 and ChEMBL412091; for series C, to the ligands of the 2VTO and 2VTQ PDB entries, and for the CDK2 series D, to 1H01 and 1OIR.

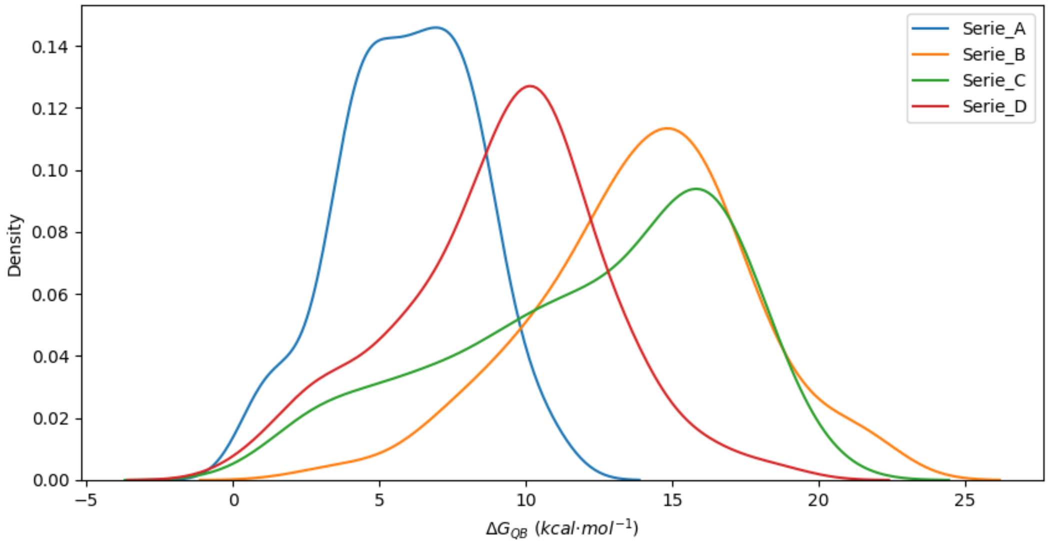

Figure S6: Distribution of ΔG_{QB} values for the four CDK2 congeneric series.

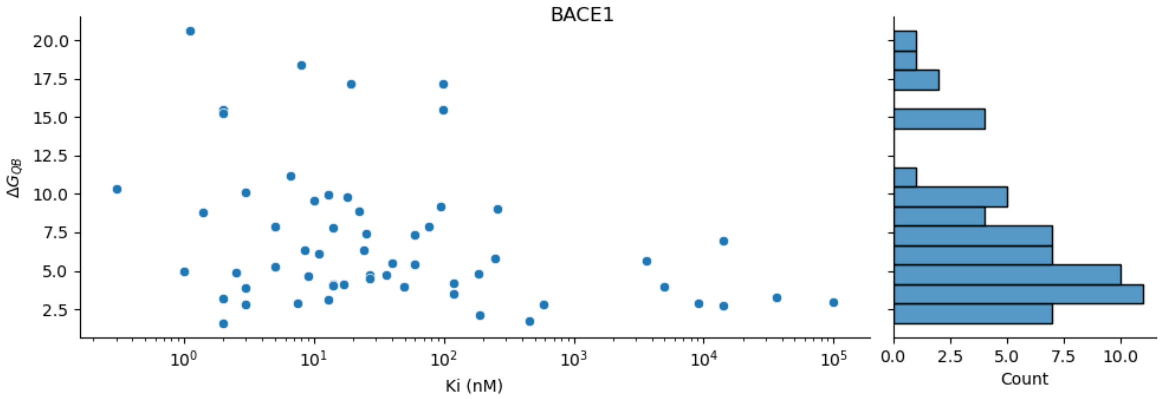

Figure S7: Relationship between K_i and predicted ΔG_{QB} of BACE1 inhibitors.

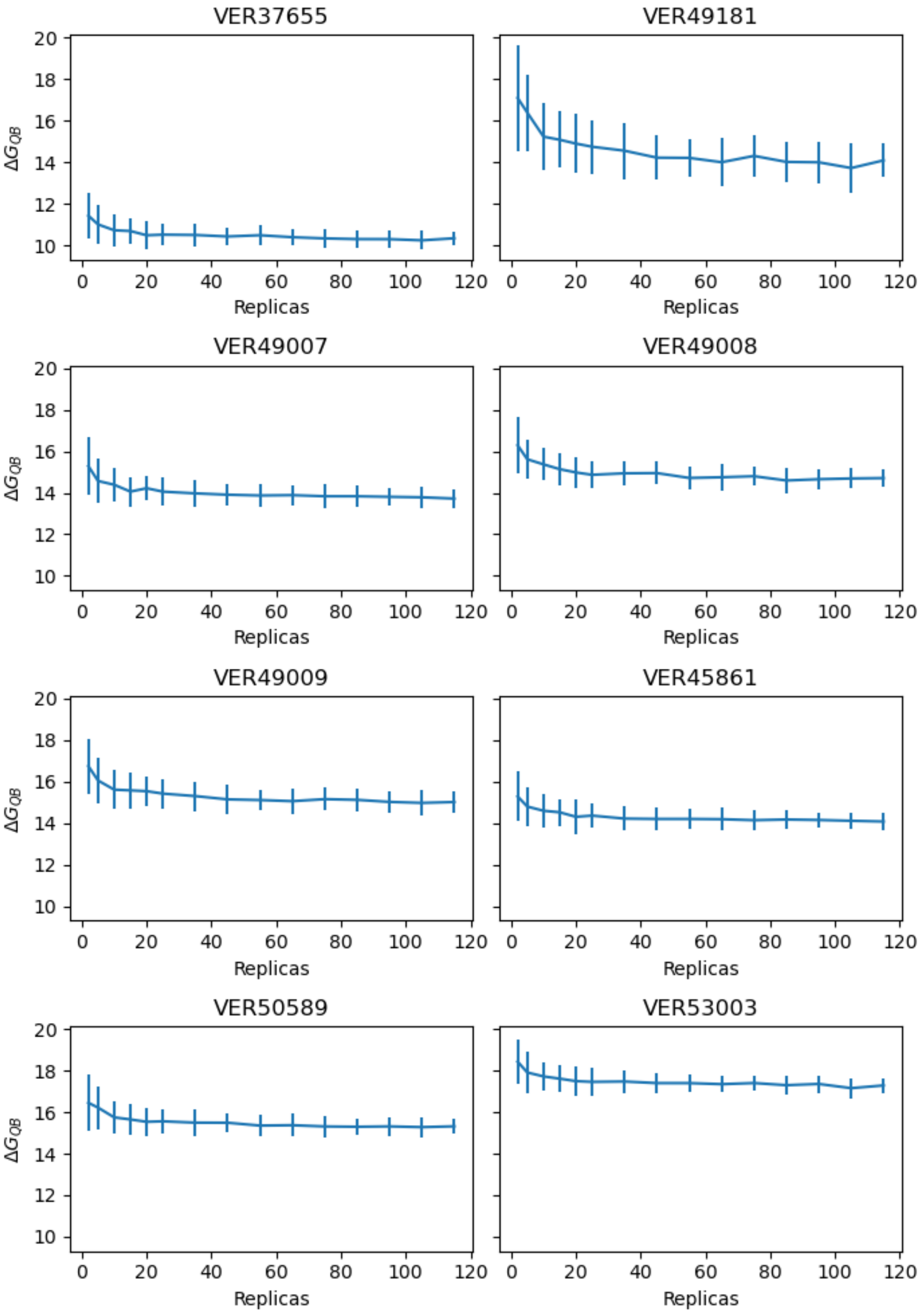

Figure S8: Convergence of the ΔG_{QB} calculations based on the amount of SMD replicas. The error bars show the standard deviation of the ΔG_{QB} distribution obtained from resampling the n replicas with replacement.

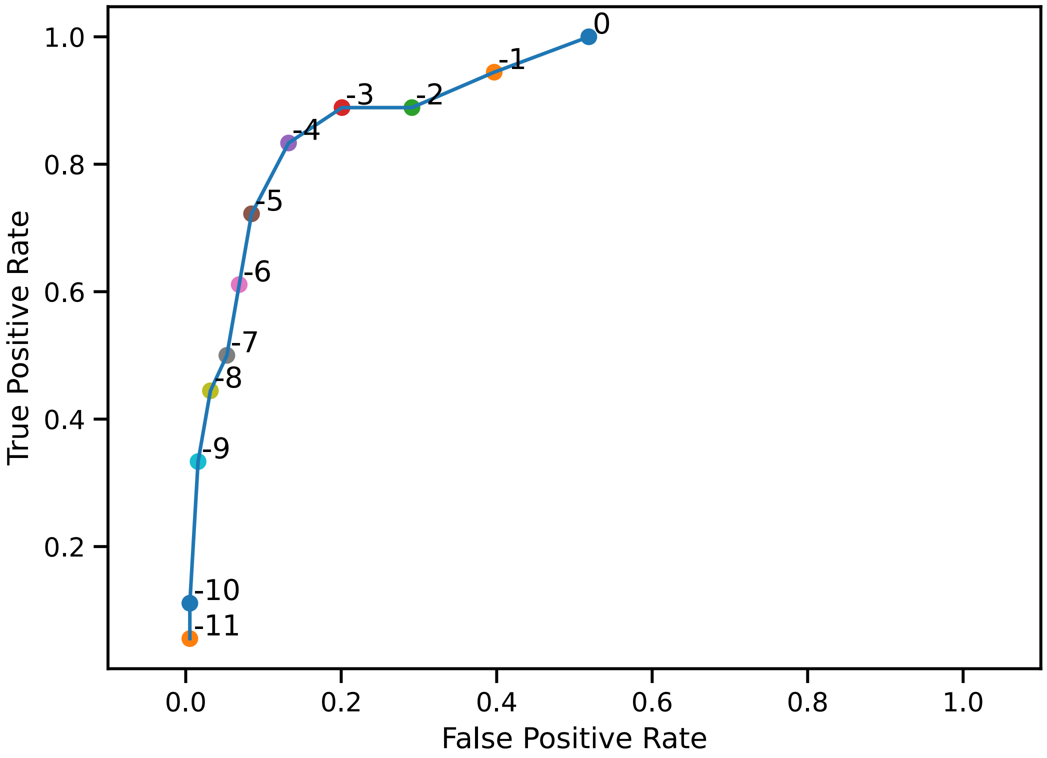

Figure S9. Receiving operating curve of the activity cliff predictions using the HSP90α dataset. Each dot represents the $\Delta\Delta G_{QB}$ threshold at which the ligand pairs are classified as activity cliffs, using kcal mol^{-1} as units.

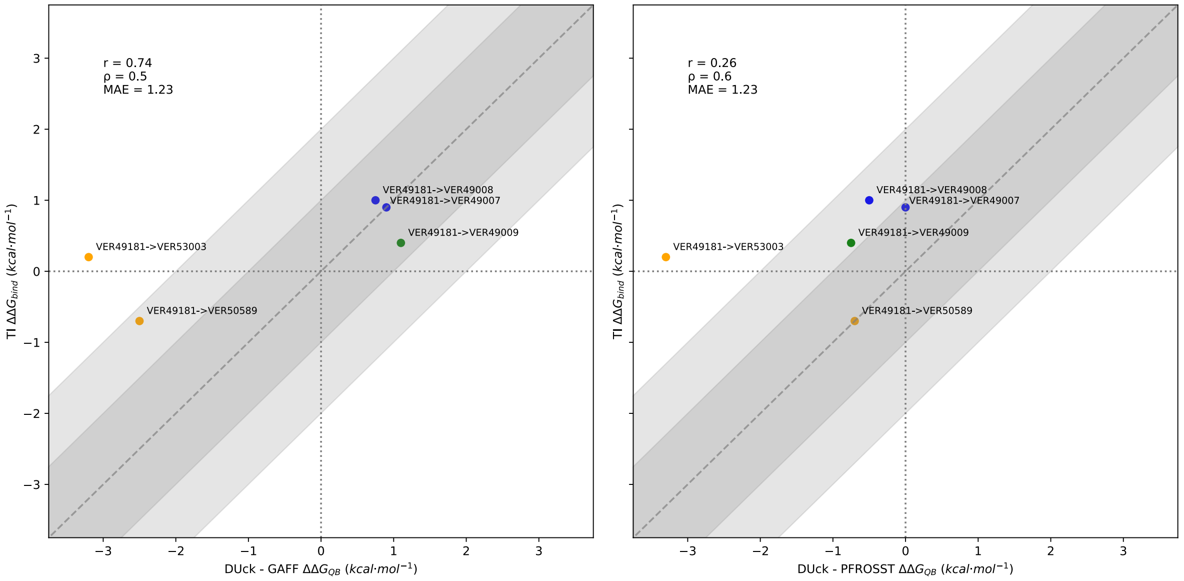

Figure S10: Correlation between $\Delta\Delta G_{QB}$ and $\Delta\Delta G_{Bind}$ predicted by TI for the Vernalis HSP90α dataset. The 5 selected ligand comparisons correspond to the transformation paths performed in TI (Figure S1), but only the end-point ligands are compared in DUck. Transformations to isoxazole, inverse isoxazole (orange circles), or pyrazoles (other colors) are in distinct areas of the Figure. One can distinguish between transformations of the core and the R-groups.

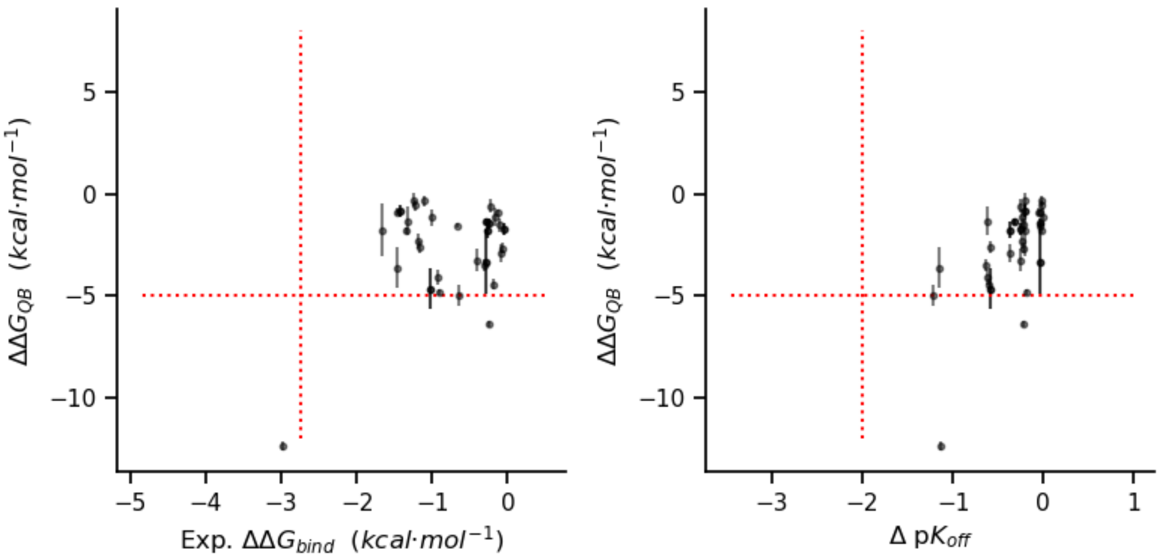

Figure S11. Prediction of MMP activity cliffs from the HSP90α Kokh et al. dataset. The matched molecular pairs were obtained using the mmpdb tool. Comparison between (left) $\Delta\Delta G_{QB}$ vs. $\Delta\Delta G_{bind}$ and (right) $\Delta\Delta G_{QB}$ vs. ΔpK_{off} for the 188 compound pairs, respectively. A $\Delta\Delta G_{QB}$ threshold of 5 kcal mol^{-1} is set for predicting the activity cliffs with a $\Delta\Delta G_{bind}$ cut-off of 2.73 kcal mol^{-1}. The error bars correspond to the sum of standard deviations for each compared pair. Both thresholds are indicated as dotted lines.
